# Complement Susceptibility in Relation to Genome Sequence of Recent Klebsiella pneumoniae Isolates from Thai Hospitals

**DOI:** 10.1128/mSphere.00537-18

**Published:** 2018-11-07

**Authors:** Jessica Loraine, Eva Heinz, Jessica De Sousa Almeida, Oleksandr Milevskyy, Supayang P. Voravuthikunchai, Potjanee Srimanote, Pattarachai Kiratisin, Nicholas R. Thomson, Peter W. Taylor

**Affiliations:** a School of Pharmacy, University College London, London, United Kingdom; b Wellcome Trust Sanger Institute, Hinxton, Cambridge, United Kingdom; c Faculty of Science, Prince of Songkla University, Songkla, Thailand; d Faculty of Allied Health Sciences, Thammasat University, Pathumtanee, Thailand; e Faculty of Medicine Siriraj Hospital, Mahidol University, Bangkok, Thailand; f London School of Hygiene and Tropical Medicine, London, United Kingdom; University of Kentucky

**Keywords:** *Klebsiella pneumoniae*, complement resistance, lipopolysaccharide, polysaccharide capsules, whole-genome sequencing

## Abstract

The capacity to resist the bactericidal action of complement (C′) is a strong but poorly understood virulence trait in *Klebsiella* spp. Killing requires activation of one or more C′ pathways, assembly of C5b-9 membrane attack complexes (MACs) on the surface of the outer membrane (OM), and penetration of MACs into the target bilayer. We interrogated whole-genome sequences of 164 *Klebsiella* isolates from three tertiary hospitals in Thailand for genes encoding surface-located macromolecules considered to play a role in determination of C′ resistance. Most isolates (154/164) were identified as Klebsiella pneumoniae, and the collection conformed to previously established population structures and antibiotic resistance patterns. The distribution of sequence types (STs) and capsular (K) types were also typical of global populations. The majority (64%) of isolates were resistant to C′, and the remainder were either rapidly or slowly killed. All isolates carried genes encoding capsular polysaccharides (K antigens), which have been strongly linked to C′ resistance. In contrast to previous reports, there were no differences in the amount of capsule produced by C′-resistant isolates compared to C′-susceptible isolates, nor was there any correlation between serum reactivity and the presence of hypermucoviscous capsules. Similarly, there were no correlations between the presence of genes specifying lipopolysaccharide O-side chains or major OM proteins. Some virulence factors were found more frequently in C′-resistant isolates but were considered to reflect clonal ST expansion. Thus, no single gene accounts for the C′ resistance of the isolates sequenced in this study.

**IMPORTANCE** Multidrug-resistant Klebsiella pneumoniae is responsible for an increasing proportion of nosocomial infections, and emerging hypervirulent K. pneumoniae clones now cause severe community-acquired infections in otherwise healthy individuals. These bacteria are adept at circumventing immune defenses, and most survive and grow in serum; their capacity to avoid C′-mediated destruction is correlated with their invasive potential. Killing of Gram-negative bacteria occurs following activation of the C′ cascades and stable deposition of C5b-9 MACs onto the OM. For *Klebsiella*, studies with mutants and conjugants have invoked capsules, lipopolysaccharide O-side chains, and OM proteins as determinants of C′ resistance, although the precise roles of the macromolecules are unclear. In this study, we sequenced 164 *Klebsiella* isolates with different C′ susceptibilities to identify genes involved in resistance. We conclude that no single OM constituent can account for resistance, which is likely to depend on biophysical properties of the target bilayer.

## Abstract (precis)

Multidrug-resistant Klebsiella pneumoniae is responsible for an increasing proportion of nosocomial infections, and emerging hypervirulent K. pneumoniae clones now cause severe community-acquired infections in otherwise healthy individuals. These bacteria are adept at circumventing immune defenses, and most survive and grow in serum; their capacity to avoid C′-mediated destruction is correlated with their invasive potential. Killing of Gram-negative bacteria occurs following activation of the C′ cascades and stable deposition of C5b-9 MACs onto the OM. For *Klebsiella*, studies with mutants and conjugants have invoked capsules, lipopolysaccharide O-side chains, and OM proteins as determinants of C′ resistance, although the precise roles of the macromolecules are unclear. In this study, we sequenced 164 *Klebsiella* isolates with different C′ susceptibilities to identify genes involved in resistance. We conclude that no single OM constituent can account for resistance, which is likely to depend on biophysical properties of the target bilayer.

## INTRODUCTION

Klebsiella pneumoniae is a prime cause of systemic nosocomial and community-acquired infections in immunocompromised individuals and, increasingly, healthy individuals (1–3). K. pneumoniae has for many years been implicated as a causative agent of pneumonia, bacteremia, wound infections, urinary tract infections, and meningitis in hospitalized patients. The therapeutic challenges posed by K. pneumoniae have been compounded by the capacity of these ubiquitous opportunistic pathogens to acquire resistance to a wide range of antibiotics, notably carbapenems and other broad-spectrum β-lactam agents (4). Resistance is also emerging against tigecycline (5) and colistin (6), drugs of last resort for the treatment of multidrug-resistant infections. In recent years, hypervirulent K. pneumoniae clones associated with pyogenic liver abscesses, pneumonia, and meningitis in younger, otherwise healthy patients have emerged; such isolates have acquired genetic traits associated with increased virulence, and while the majority are currently susceptible to antibiotics, drug-resistant hypervirulent isolates are beginning to emerge (1, 3). The trend toward untreatable, invasive infections shows no signs of abating (7, 8), and new therapies are badly needed to extend treatment options for these life-threatening infections.

The targeting of bacterial determinants required for virulence is a chemotherapeutic approach that is gathering interest, and there is some evidence that inhibiting the expression of key virulence factors can resolve bacterial infections in animal models (9–11). K. pneumoniae characteristically produces copious amounts of capsular polysaccharide, and these K antigens are well-established virulence factors contributing to invasive disease (1, 12). However, relatively few other K. pneumoniae virulence determinants have been implicated in systemic infection, although lipopolysaccharide (LPS) O-side chains, iron acquisition systems such as siderophores, and adhesins, which vary in frequency among clinical isolates, contribute to disease severity in animal models of infection and are common in hypervirulent isolates (1, 8, 13, 14). The complement (C′) system is a first line of defense against systemic invasion by microbial intruders that have penetrated the host’s epithelial barriers, and evasion of the C′ system greatly enhances the capacity of Gram-negative pathogens to survive and multiply in blood and in the major organs (15). Hypervirulent K. pneumoniae clinical isolates belong predominantly to capsule serotype K1 and to a lesser extent K2 (ST23 and ST86; 16) and tend to be refractory to the bactericidal action of complement (17). Loss by mutation of *wzy*_K1 (previously *magA*), the serotype K1 capsule polymerase gene (18), transformed hypervirulent strains to extreme C′ susceptibility, strongly implicating the capsule as a determinant of C′ resistance (17).

A comprehensive understanding of the mechanisms of K. pneumoniae C′ resistance, which is currently lacking, is likely to enable identification of additional targets for therapeutic intervention or new strategies for augmentation of host defenses. A limited number of enterobacteria avoid C′-mediated attack by preventing activation of all three C′ pathways. Bacterial activation of the classical, lectin, or alternative pathways results in covalent binding of C3 cleavage products to the bacterial surface, formation of a C5 convertase and generation of the C5b-9 membrane attack complex (MAC) (19, 20). Following cleavage of C5, each molecule of the larger fragment C5b initiates assembly of a MAC by recruiting single molecules of C6, C7, and C8; multiple copies (up to 18) of C9 join the membrane-embedded C5b-8 assembly to form the membrane-perturbing MAC. Intercalation of C5b-9 containing at least two copies of pore-forming C9 into lipid domains of the outer membrane (OM) results in killing of susceptible bacterial targets (21, 22). Thus, enterobacterial C′ resistance is usually due to the inability of C5b-9 complexes to assemble at the bacterial surface or insert in stable fashion into the OM, with the consequence that no C5b-9 can be found in stable association with this bilayer (19–21). Capsules and long and numerous LPS O-side chains undoubtedly play a role in determining resistance by preventing C′ activation or access of C′ components to the surfaces of Gram-negative bacteria, including K. pneumoniae (23), although nonencapsulated forms may be resistant to C′ and encapsulated forms may be susceptible to C′ (19).

While not yet proven, it may be that the architecture of the external surface of the OM strongly influences the capacity of pore-generating C9 to perturb the integrity of the OM. Thus, the surfaces of C′-susceptible strains may contain sufficient numbers of exposed lipid domains to facilitate C5b-9 generation and penetration, whereas the spatial and temporal organization of the OM of resistant bacteria may be dominated by recently identified supramolecular protein assemblages (24) to a degree where there are insufficient hydrophobic domains to act as C5b-9 assembly and binding sites. OM proteins, such as plasmid-encoded TraT (25) or bacteriophage-derived Iss (26) and Bor (27), have been implicated as determinants of C′ resistance, but these studies employed gene transfer into C′-susceptible genetic backgrounds, leading to insertion into the OM bilayer of protein copy numbers (>20,000 molecules per cell) far in excess of those found naturally. Insertion of large numbers of protein molecules into the OM will almost certainly alter the biophysical properties of the bilayer, reducing the surface area and fluidity of lipid patches that are essential for binding and assembly of the MAC. Similarly, identification of OM proteins contributing to C′ resistance by gene deletion risks disrupting the integrity of the OM, enabling C5b-9 insertion and membrane perturbation, and may be unrelated to any functions ascribed to such proteins; complementation simply restores the functional integrity of the OM. In the study reported here, we have adopted a different approach: we determined whole-genome sequences of 164 recent C′-susceptible and C′-resistant *Klebsiella* isolates from three tertiary care hospitals in Thailand and probed the sequence data for correlates to C′ reactivity. We determined that the amount of capsule produced, hypermucoviscosity, and the presence of genes encoding LPS O-side chains, the major OM proteins, and virulence determinants were unable to explain the reactivity to human serum C′ of the isolates.

## RESULTS

The genomes of 164 presumptive Klebsiella pneumoniae isolates derived from blood, urine, pus, sputum, and ascitic fluid samples by routine culture from three hospitals in Thailand were sequenced; 30 isolates were from Thammasat University Hospital, Pathum Thani Province, 89 isolates were from Siriraj Hospital, Bangkok, and 45 isolates were from Songklanagarind Hospital, Hat Yai, Songkhla Province (see Data Set S1 in the supplemental material). Siriraj is the largest hospital in Thailand with 2,300 beds, 1,000,000 outpatients per annum, and 80,000 inpatients per annum; equivalent figures for Songklanagarind are 846, 1,019,375, and 40,936, respectively, and for Thammasat, 601, 384,088, and 40,745, respectively (all data from 2017).

10.1128/mSphere.00537-18.1 DATA SET S1 *Klebsiella* isolates used in this study. Download Data Set S1, XLSX file, 0.03 MB. Copyright © 2018 Loraine et al. 2018 Loraine et al. This content is distributed under the terms of the Creative Commons Attribution 4.0 International license.

**FIG 1:**
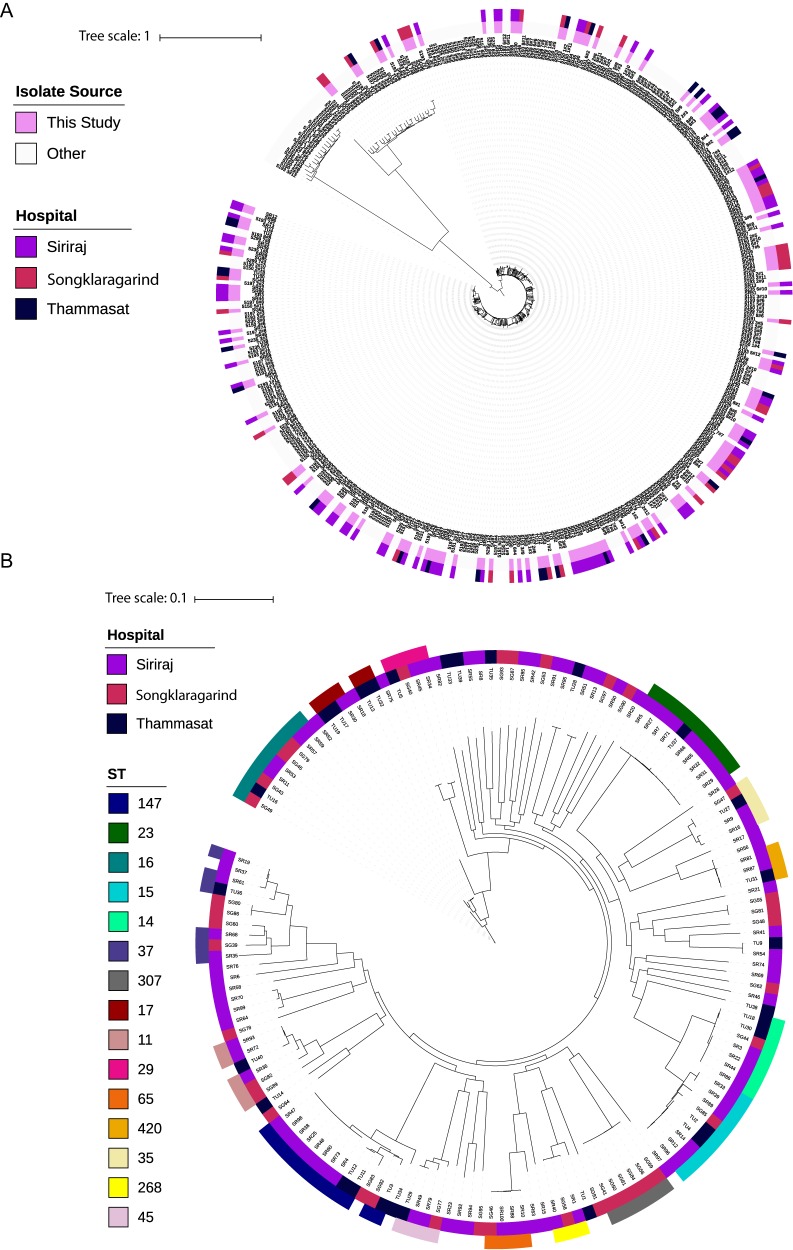
Population structure of *Klebsiella* clinical isolates. (A) Phylogenetic tree based on the core gene SNP alignment of 164 Thai *Klebsiella* genomes, 247 genomes from the global K. pneumoniae collection (7). (B) Phylogeny of core gene SNPs from 154 K. pneumoniae Thai isolates. Isolates from Tammasat University Hospital, Siriraj Hospital, and Songklanagarind Hospital are designated TU, SR, and SG, respectively; details can be found in Data Set S1 in the supplemental material. Sequence types (STs) are shown as indicated in the legend.

The large majority of isolates (154 of 164) were identified as K. pneumoniae *sensu stricto*, the species most closely associated with human infection (7). The remaining 10 isolates belonged to the species Klebsiella quasipneumoniae, which is part of the K. pneumoniae species complex comprising K. pneumoniae *sensu stricto*, K. quasipneumoniae, and K. variicola. All show the same clinical manifestation and are routinely diagnosed as K. pneumoniae. Phylogenetic analysis of our data in the context of a global collection (7) demonstrates that the Thai collection is representative of the global population structure (Fig. 1A). Core gene SNPs were used to determine the population structure of the K. pneumoniae Thai isolates (Fig. 1B). The phylogenetic tree supports the deep-branching, star-like population structure proposed by Holt et al. (7) for K. pneumoniae that indicates early radiation into a large number of distinct, equally distant lineages. The most common sequence types (STs) were ST147 (7.3%), ST23 (6.1%), ST16 (5.5%), and ST15 (5.5%); other STs each accounted for <5% (Fig. 1B). European and Asian isolates of ST147 and ST15 are characterized by multidrug resistance (2, 28, 29). Interestingly, ST16 is associated with sporadic infections in the United Kingdom and southern Europe (2, 30). ST23 isolates often display the hypervirulent phenotype that is strongly associated with community-acquired liver abscesses in the Far East (31) and produce a hypermucoviscous K1 capsule linked to C′ resistance (17).

### Presence of antibiotic resistance genes.

Acquired antimicrobial resistance (AMR) genes were determined with the curated AMR database tool ARG-ANNOT using Ariba. The core chromosomal *SHV* (β-lactamase) and *oxqAB* (conferring low-level resistance to quinolones) genes were found in 100 and 98% (164 and 161 isolates, respectively) of the Thai isolates. The proportion of K. pneumoniae isolates carrying acquired AMR genes (Fig. S1) was generally comparable to the global pattern established by Holt et al. (7). The rifampin resistance gene *arr* was present in 18% (27/154) of isolates, a similar incidence to that reported in Vietnamese isolates (7); *arr* was, however, enriched in the Songklanagarind isolates compared to those from Siriraj and Thammasat, with 40% (15/38) carrying this gene. The quinolone resistance gene *qnrB* was also found more frequently in Songklanagarind isolates than those from the other two hospitals (29% compared to 14% overall; 12/38 compared to 22/154), and the frequency of isolation was higher than that reported for the global collection (7). The majority (95% [36/38]) of Songklanagarind isolates carried *bla*_CTX-M_ genes compared to 48% (72/154) overall in the Thai collection; *tetA* (71% [27/38]; 39% [60/154] overall) was also enriched in Songklanagarind isolates. Thammasat isolates carried fewer AMR genes compared to isolates from Songklanagarind and Siriraj. NDM-1 carbapenemase was found in 8% (7/88) of Siriraj isolates and in ST14-16 lineages. PlasmidFinder revealed the presence of 39 previously identified plasmid replicons in 146 of the 164 Thai isolates.

10.1128/mSphere.00537-18.2 FIG S1 Presence of genes encoding antibiotic resistance in Thai K. pneumoniae isolates. AGly, aminoglycosides; Bla, β-lactamases; Rif, rifampin; Phe, chloramphenicol; Tmt, trimethoprim; MLS, macrolides; Flq, fluoroquinolones; Sul, sulfonamides; Tet, tetracycline. AMR genes were sourced from the curated version of the ARG-ANNOT database available at the SRST2 site (84). Download FIG S1, EPS file, 6.3 MB. Copyright © 2018 Loraine et al. 2018 Loraine et al. This content is distributed under the terms of the Creative Commons Attribution 4.0 International license.

### C′ susceptibility of K. pneumoniae isolates.

**FIG 2:**
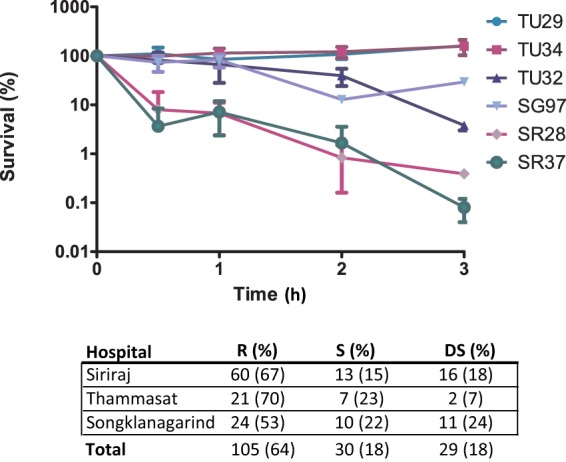
Susceptibility of 164 Thai K. pneumoniae/K. quasipneumoniae isolates to the C′-mediated bactericidal action of pooled normal human serum. Isolates were classified as C′ resistant (R) (no reduction in viable count during the 3-h incubation period), delayed susceptible (DS) (>10% survival after 1-h incubation, <90% after 3-h incubation), or rapidly susceptible (S) (<10% after 1-h incubation) (the percentage of the total from each hospital is shown at the bottom of the figure). Two examples of each category are shown, and all determinations were performed at least twice on different days. The TU29 and TU34 isolates are C′ R, the SG97 and TU32 isolates are C′ DS, and the SR28 and SR37 isolates are C′ S.

The method employed to determine susceptibility to pooled human serum (32) enabled the isolates to be assigned to one of three categories: resistant (R), delayed susceptible (DS), and rapidly susceptible (S). Examples of these serum responses are shown in Fig. 2. R isolates showed no reduction in viable count during the 3-h incubation period, DS isolates displayed >10% survival after 1-h incubation and <90% after 3-h incubation, and the inoculum of S isolates was reduced to <10% after 1-h incubation. C′-susceptible enterobacteria that express long and numerous LPS O-side chains (smooth phenotype) typically exhibit a delayed response of at least 1 h, whereas strains lacking the O-side chain moiety of LPS (rough phenotype) are usually promptly killed by C′, although occasional clinical isolates with rough phenotype exhibit delayed serum sensitivity due to the presence of a polysaccharide capsule (33). Fully C′-resistant isolates are invariably of the smooth phenotype (19, 34). Susceptibility of all 164 *Klebsiella* isolates to human serum is summarized in Fig. 2. The majority (64%) were complement resistant; susceptible isolates were equally split between DS and S phenotypes.

### Relationships between capsules, presence of LPS O-side chain genes, and reactivity of K. pneumoniae to human serum.

Sequencing of K. pneumoniae clinical isolates has revealed that the pathogen produces few virulence factors that specifically target the host’s tissues or immune system (3). Rather, K. pneumoniae has adopted a strategy of navigation and negation of host immune defenses mediated by capsule, LPS, fimbriae, siderophores, urease, and efflux pumps to protect against phagocytosis, antimicrobial peptides, and C′-mediated killing (1). Capsules have received a great deal of attention as key surface determinants contributing to C′ resistance, as poorly encapsulated and nonencapsulated mutant bacteria appear to bind more C3 than K. pneumoniae clinical isolates displaying more extensive capsules (35, 36); loss of capsular polysaccharide sometimes (17) but not always (37, 38) leads to increased susceptibility to C′. In addition, capsular hypermucoviscosity is correlated with C′ resistance in liver-invasive strains (17), and the presence of sialic acid as a capsular component of hypervirulent K. pneumoniae (39) may reduce C′ susceptibility by facilitating the binding of factor H to C3b to prevent activation of the alternative pathway (40). We determined the capsular (K) and LPS O-side chain (O) serotypes by *in silico* typing (41, 42) and investigated the presence of genes conferring the capacity to synthesize sialic acid. Capsule hypermucoviscosity was determined using the string test (17) and correlated with the presence or absence of *rmpA*. Capsule surface area was measured by light microscopy in order to examine K. pneumoniae isolates for correlates with C′ susceptibility.

**FIG 3:**
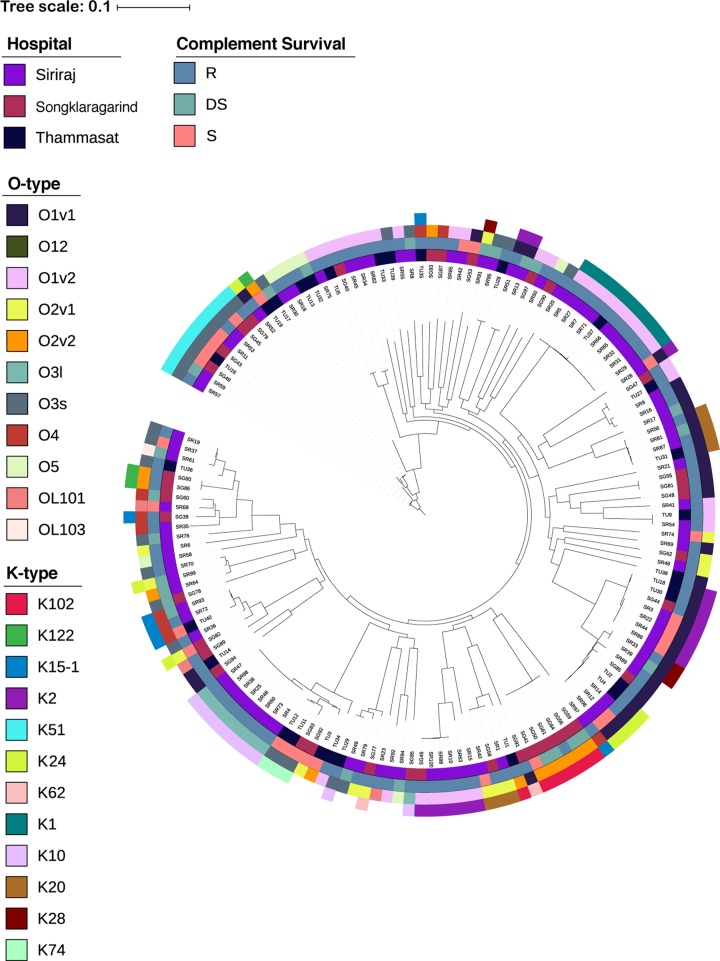
Capsular (K) and LPS O antigen *in silico* typing of Thai K. pneumoniae isolates. The phylogenetic tree is as shown in Fig. 1B. The K and O types as well as C′ susceptibility and isolate source are shown as indicated in the figure.

All K. pneumoniae isolates were encapsulated, as determined by the presence of K-antigen biosynthesis gene clusters (Fig. 3) and by India ink negative staining (Fig. S2). In accord with other studies (7, 41, 42), there was a high degree of diversity of K serotypes. Fifty-nine distinct K types were represented with two isolates of unknown, probably novel, K type; K2 (10.4%), K51 (7.1%), K1 (6.5%), K10 (6.5%), K20 (4.5%), and K24 (4.5%) were frequently encountered, and some differences between sources within Thailand were evident (Fig. 3 and Fig. S2). For example, K1 and K2 were common in Siriraj isolates, and K102 represented a larger proportion of Songkhlanagarind isolates compared to the other two sources. The wide range of K types was distributed among a relatively small number (nine) of O types (Fig. 3 and Fig. S3). All K1 (all ST23) and K10 (eight ST147, one each of ST45 and ST629) isolates were C′ resistant (both 10/10), whereas C′-susceptible isolates were well represented among K2 (6/16), K51 (6/11), and K24 (3/7) isolates. All three K74 isolates (two ST147 isolates and one ST273 isolate) were sensitive to C′ (Table S1).

10.1128/mSphere.00537-18.3 FIG S2 Visualization of K. pneumoniae capsules by India ink negative staining and transmitted light microscopy. Representative images of C′ R isolates TU29 and TU34 (panels A and B, respectively), DS isolates TU32 and SG97 (panels C and D, respectively), and S isolates SR28 and SR37 (panels E and F, respectively) are shown together with K. pneumoniae isolate B5055 and its capsule-negative derivative B5055nm derived by gene knockout (panels G and H, respectively). Strain B5055nm served as a negative control. Download FIG S2, TIF file, 9.2 MB. Copyright © 2018 Loraine et al. 2018 Loraine et al. This content is distributed under the terms of the Creative Commons Attribution 4.0 International license.

10.1128/mSphere.00537-18.4 FIG S3 Capsular (K) and LPS O serotypes in Thai K. pneumoniae isolates. Distribution of major capsule (A) and O (B) types. (C) Combinations of O and K antigens within the Thai isolate collection. Download FIG S3, TIF file, 9.7 MB. Copyright © 2018 Loraine et al. 2018 Loraine et al. This content is distributed under the terms of the Creative Commons Attribution 4.0 International license.

10.1128/mSphere.00537-18.7 TABLE S1 Relationship between capsular (K) serotype and susceptibility to C′. R, no reduction in viable count during the 3-h incubation period; DS (delayed susceptible), >10% survival after 1-h incubation and <90% survival after 3-h incubation; S (rapidly susceptible), <10% survival after 1-h incubation. Download Table S1, DOCX file, 0.01 MB. Copyright © 2018 Loraine et al. 2018 Loraine et al. This content is distributed under the terms of the Creative Commons Attribution 4.0 International license.

**FIG 4:**
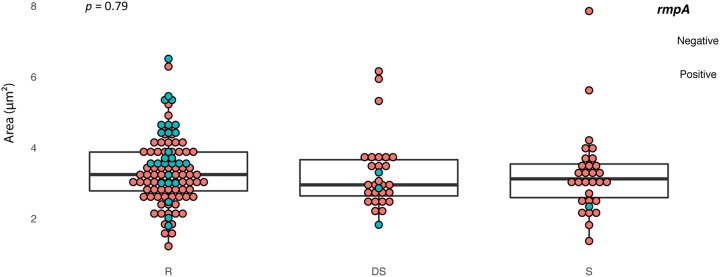
Capsule size of *Klebsiella* isolates in relation to C′ susceptibility. Distribution of capsule size between groups based on C′ reactivity. The area (μm^2^) occupied by capsule for each isolate was determined by CellProfiler image analysis of 40 to 100 individual bacteria. R, C′ resistant; DS, C′ delayed susceptible; S, C′ rapidly susceptible. *P* values were determined by ANOVA. The lower and upper hinges of the boxplots correspond to the first and third quartiles (25th and 75th percentiles). Isolates carrying *rmpA* (regulator of the mucoid phenotype A gene) are indicated.

The size of the capsule for each isolate was determined by negative imaging with India ink. Calculation of the area occupied by the capsule was determined from micrographs using CellProfiler image analysis software (Fig. 4); 40 to 100 cells were measured in each preparation, and differences between C′-resistant and -susceptible groups were compared by ANOVA. There was some variation in capsule size within each sample, but no significant differences (*P* = 0.79; resistant versus susceptible) in mean capsule area between C′-resistant (mean, 3.36 ± 0.94 μm^2^), S (3.23 ± 1.43 μm^2^), and DS (3.26 ± 1.06 μm^2^) isolates (Fig. 4). The presence of a hypermucoviscous capsule was identified by formation of viscous strings >5 mm in length when stretched from a colony on a sheep blood agar plate (17). We examined Thai K. pneumoniae isolates for hypermucoviscous capsules following overnight growth on sheep blood agar. There was no significant relationship between C′ reactivity and hypermucoviscosity: 48/105 isolates were C′ resistant, 13/29 were DS, and 19/30 S isolates were string test positive (χ^2^ test of independence, χ^2^ 3.1194, *P* 0.21). The hypermucoviscosity trait was associated with most of the major K types in the collection, and all but one K. pneumoniae K1 isolate were string test positive. There was a strong association (28/30) between a positive string test and the presence of *rmpA*; this gene regulates the mucoid phenotype by activating capsule production (31, 43). The uronic acid component of capsular polysaccharides from selected strains was estimated by using the sodium tetraborate reaction as an alternative chemical method to estimate capsule content. Partially purified capsular polysaccharides from logarithmic phase cultures of 10 isolates belonging to each susceptibility group were examined. Isolates were selected to cover the range of capsule areas as determined by negative staining: as with the India ink method, there were no significant differences in the amount of uronic acid associated with the capsule extracts (Fig. S4).

10.1128/mSphere.00537-18.5 FIG S4 Capsule content of ten logarithmic-phase K. pneumoniae isolates from each C′ susceptibility group determined by partial capsule extraction followed by the sodium tetraborate reaction (90, 91) to estimate uronic acid content. Assays were performed twice and with duplicate samples for each isolate. The lower and upper hinges of the boxplots correspond to the first and third quartiles (25th and 75th percentiles). Download FIG S4, TIF file, 6.3 MB. Copyright © 2018 Loraine et al. 2018 Loraine et al. This content is distributed under the terms of the Creative Commons Attribution 4.0 International license.

Of genes and gene products associated with sialic acid synthesis and polymer export in Escherichia coli (44), only NeuB has been found in K. pneumoniae (UniProt accession no. A0A1C3SZN5). We derived the DNA sequence of this protein and screened the Thai K. pneumoniae sequences with Ariba for evidence of *neuB*: none was found. In contrast, *nanT*, encoding a sialic acid transport protein associated with intracellular catabolism and inducible by the substrate (44), was present in 161 of 164 *Klebsiella* genomes.

### Relationship between OM proteins and C′ susceptibility.

Two major *Klebsiella* OM proteins, OmpK35 and OmpK36, are closely associated with antibiotic resistance (45, 46). There is no publicly accessible *Klebsiella* OM protein database. We therefore constructed customized Ariba gene databases for these two proteins and for LppA, Pal, and OmpK17; these three proteins have been linked to C′ resistance in K. pneumoniae, *Salmonella enterica* serotype Typhimurium, and other enterobacteria (47, 48). *ompK35* and *ompK36* were detected in all 164 Thai isolates. In 21 isolates, *ompK35* was either fragmented or interrupted, but there was no association between these isolates and C′ resistance. *ompK17* and *pal* were present in all 164 strains, and *lppA* was present in all but four isolates. Therefore, no clear associations between genes encoding these proteins and susceptibility to C′ were evident. However, in order to fully investigate potential associations, a comprehensive curated OM protein database and monitoring of gene expression will be required.

### Virulence determinants of K. pneumoniae isolates.

**FIG 5:**
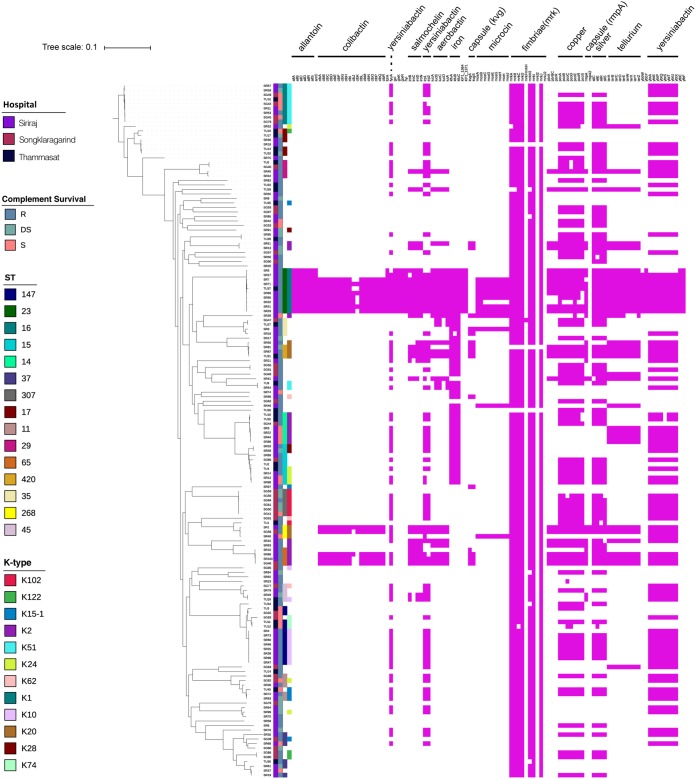
Virulence genes associated with Thai K. pneumoniae isolates. The guidance tree is as shown in Fig. 1B. Virulence genes were predicted using Ariba based on the BIGsDB virulence genes described for K. pneumoniae (7).

Iron-sequestering systems have been implicated in the determination of C′ resistance through their role in metabolic adaptation (49, 50) and are considered major virulence effectors in *Klebsiella* infections (1, 7, 51). We examined the distribution of established virulence genes (7, 52) among the 154 K. pneumoniae Thai isolates (Fig. 5). The iron-sequestering siderophores aerobactin, salmochelin, and yersiniabactin were widely distributed among these isolates. C′-resistant ST23 K1 serotype isolates were enriched for colibactin, microcin, and other virulence genes compared to isolates from other STs and K serotypes. Such isolates are strongly associated with highly invasive infections (7). As expected (1, 7, 52), virtually all isolates carried genes for elaboration of fimbriae. Genes encoding components involved in metabolism of allantoin, enabling utilization of this metabolite under aerobic conditions, were restricted to ST23 K1 isolates; hypervirulent K. pneumoniae use this capacity to enhance virulence (1). Although colibactin genes were associated exclusively with C′-resistant isolates, they were carried by only a small number of isolates. Other virulence determinants were distributed throughout C′-resistant, DS, and S groups. There were no clear associations between C′ resistance and any one set of virulence genes (Fig. S5).

10.1128/mSphere.00537-18.6 FIG S5 Incidence of virulence genes in K. pneumoniae. Download FIG S5, EPS file, 1.5 MB. Copyright © 2018 Loraine et al. 2018 Loraine et al. This content is distributed under the terms of the Creative Commons Attribution 4.0 International license.

## DISCUSSION

Both humoral and cellular defenses are considered important for prevention of tissue and blood invasion by *Klebsiella* spp. Although K. pneumoniae is considered to be a predominantly C′-resistant pathogen (1), there have been surprisingly few studies delineating the degree of C′ resistance among clinical isolates. Recent evidence has emphasized that survival of K. pneumoniae in blood is, along with a capacity to counter phagocytosis by macrophages and neutrophils, a critical virulence trait associated with systemic invasion (53, 54). Indeed, neutrophils may aid the dissemination and establishment of secondary sites of infection by hypervirulent K. pneumoniae (55). Sahly and coworkers (56) examined the serum susceptibility of an international collection of nosocomial K. pneumoniae isolates in relation to extended-spectrum β-lactamase (ESBL) production. After they excluded clonal strains, they found that 36% (17/47) of ESBL producers and 16% (27/166) of non-ESBL producers were C′ resistant (56), suggesting a greater pathogenic potential for ESBL-producing isolates. In another study, six ST258 clinical isolates displayed a wide range of responses to C′ in whole human blood (53), emphasizing a more complex association between invasive potential and C′ susceptibility than previously recognized. In the current study, the majority (64%) of isolates were fully resistant to C′, but the relative abundance of susceptible isolates provided an opportunity to examine the basis of differences in C′ reactivity of K. pneumoniae without resort to the generation of mutants lacking key surface components.

Prevention of C′ activation or the failure of MACs to insert into the target OM bilayer of Gram-negative bacteria is a reflection of the distribution of macromolecules at the bacterial surface. A limited number of studies have shown that both C′-resistant and C′-susceptible strains of K. pneumoniae are able to activate the classical and alternative pathways to various degrees (53, 57, 58), but there has been no systematic evaluation of relationships between the degree of activation, deposition onto the surfaces of key proteins such as C3b and C5b-9, and bacterial killing or of the capacity of major surface components such as capsule, LPS O-side chains, and proteins to modulate activation. The recent recognition that K. pneumoniae capsules may incorporate sialyl residues (39, 59), with the potential to prevent activation of the alternative pathway (40), would make such an analysis a key to understanding C′ resistance mechanisms in this bacterial species.

Capsules are considered likely determinants of C′ resistance in K. pneumoniae (1, 23), as they function as macromolecules that reduce binding to the surfaces of key components of the cascade such as C3b (35, 60) and prevent MAC formation or insertion into the OM bilayer (17, 61). Most importantly, selective removal of the polysaccharide capsule using bacteriophage-associated depolymerases increases C′ susceptibility and enhances survival of K. pneumoniae-infected mice (62–64). However, we found no evidence that differences in the quantity or viscosity of capsule produced by isolates from our Thai collection could be responsible for their reactivity in serum. As anticipated, we encountered a wide range of STs and K types, and although isolates from the same clonal lineages, such as the ST23 K1 serotype isolates (SR5 through SR29; Fig. 3), tended to respond to exposure to C′ in a similar fashion, the limited numbers of isolates in each ST or K serogroup were too low to draw firm conclusions with regard to differences in serum susceptibility between lineages and capsule types. However, the basis of C′ resistance in Gram-negative bacteria is related to the biophysical nature of the target for C′ deposition, the OM, and to the capacity of structures beyond the OM surface to prevent C′ activation, not to the chemical nature of surface macromolecules (15, 19, 21). The major K. pneumoniae chemotypes do not prevent C′ activation (1, 23, 37), and the current study shows that the most common K, O, and ST groups contain both C′-resistant and -susceptible isolates (Fig. 3; see also Table S1 in the supplemental material). Determination of C′ surface binding and deposition on isolates belonging to such categories but displaying different serum reactivity would resolve this limitation of the current study.

Overall, the amount of capsule produced was remarkably similar between C′-resistant and -susceptible isolates (Fig. 4), regardless of whether chromosomal *rmpA* was present. *rmpA* was present in only 30 of the Thai isolates, including all isolates of the ST23 K1 clonal lineage, and these isolates did not produce more capsule as determined by negative staining than the *rmpA*-negative isolates did. Some C′-susceptible isolates carried *rmpA*. It is unclear why the *rmpA*-positive isolates did not produce increased amounts of capsule; Cheng et al. (43) showed that deletion of *rmpA* in a K. pneumoniae K2 strain resulted in formation of small colonies with significantly reduced capsule viscosity. Viscosity could be restored by gene complementation but only in tandem with *rcsB*, suggesting that cooperation between RmpA and the cytoplasmic response regulator RcsB is required for regulation of capsule expression.

It is contended that enhanced capsule production mediated by RmpA results in the hypermucoviscosity phenotype (31, 43); we show a strong association (28/30) between *rmpA* carriage and hypermucoviscosity as determined by the string test after growth on blood agar. In solution, viscosity is influenced by polymer composition, molecular weight, concentration, and internal friction between the randomly coiled and swollen macromolecules and surrounding solvent molecules (65). The relationships between these parameters are inevitably more complex and difficult to control if the polymer is present within a bacterial colony on an agar plate, as in the string test; *rmpA*-mediated increases in the amount of polymer produced may not necessarily lead to increases in viscosity if there is no concomitant increase in the surrounding solvent water. Heating (95°C for 30 min) of heat-stable capsule from hypermucoviscous K. pneumoniae KP-M1 significantly reduced the mucoviscosity of the polymer (39), suggesting that the high viscosity of capsules from such strains could be due to polysaccharide-protein complexes that are disrupted by heat rather than being related directly to polymer concentration. This possibility would seem worthy of investigation. We obtained no genomic evidence for sialic acid synthesis in any isolates: in view of the chemical diversity of sialic acid polymer building blocks and the consequent likelihood of orthologs and paralogs of the *neu* operon genes in species other than E. coli (66), we have initiated a biochemical rather than genomic search for evidence of capsule modification with sialyl residues among these isolates, which will be reported at a later date.

Genes involved in the synthesis and assembly of LPS O-side chains were found in all isolates from our Thai collection, regardless of their response to exposure to C′. In other enterobacteria, O-side chains have been shown to increase the length of time before initiation of C′-mediated cell death (the delayed response), but alone the side chains do not account for complete C′ resistance (19, 34, 67). This may not be the case with the K. pneumoniae isolates, although the presence of these genes does not provide information on the degree of substitution of the LPS core with O-side chains, a factor known to be important in determining serum reactivity (67). C′-resistant K. pneumoniae mutants lacking O-side chains were susceptible to serum (37), and it can be surmised that, as with other enterobacteria, K. pneumoniae LPS with a high degree of substitution of core with O-side chains is necessary but not sufficient to confer full C′ resistance (19).

Overall, the percentage of strains in our collection carrying acquired AMR genes fitted well with previous findings (7). In Thailand, isolates obtained from Songklanagarind carried a higher proportion of these genes compared to isolates from Siriraj and Thammasat. In general, K. pneumoniae isolates tend toward either hypervirulence or multidrug resistance, although there have been recent reports of multidrug-resistant hypervirulent clones (68). Notably, the hypervirulent ST23 Siriraj blood isolate KP29 contained genes encoding multiple virulence determinants and four aminoglycoside-encoding genes, as well as *bla*_TEM_, *dfrA*, *mphA*, *sul1*, *sul2*, *tetA*, and *tetR* (Fig. 5 and Fig. S1).

Variations in carriage of genes encoding aerobactin, salmochelin, and yersiniabactin, enabling iron assimilation, were evident between clonal lineages; all three systems were found in ST23 K1 serotype isolates, which were almost exclusively C′ resistant, but this association is unlikely to be directly related to their serum reactivity. Efficient iron sequestration is essential for survival and growth in serum; deletion of *fur*, encoding the master regulator of the serum-induced transcriptional response, completely abrogates C′ resistance (69), highlighting the importance of metabolic competence to C′ survival. We found little or no variation in genes for the major OM proteins and no clear relationship between antibiotic susceptibility patterns and susceptibility to C′. It has been reported that C′ resistance in K. pneumoniae is correlated with production of ESBLs (57). K. pneumoniae and other Gram-negative bacteria producing ESBLs and carrying efflux pumps have altered OM surface protein expression compared to their antibiotic-susceptible counterparts (70, 71), and these changes are likely to alter, perhaps in subtle ways, the biophysical properties of the bilayer; this is turn may affect the capacity of the C′ components to bind to or insert into the bilayer. Thus, antibiotic resistance machineries may have the capacity to alter susceptibility to C′ in ways unrelated to their specific function.

The data we and others have generated can be best reconciled by consideration of the biophysical properties and surface architecture of the OM as a whole rather than by invocation of “C′ resistance genes” as has been frequently attempted in the past. C′ resistance is intimately linked to the capacity of structures at the bacterial surface to either modulate activation of the C′ cascades or prevent the stable deposition of MACs. At the present time, we lack sufficient understanding of the relationships between K. pneumoniae surface topography, activation of each C′ pathway, and the factors governing C5b-9 intercalation into the highly asymmetric OM to be able to define the mechanistic basis of C′ resistance for *Klebsiella* and other Gram-negative bacteria.

## MATERIALS AND METHODS

### Bacterial isolates and genome sequencing, assembly, and annotation.

A total of 185 K. pneumoniae isolates were cultured from blood, urine, pus, sputum, and ascitic fluid samples at the clinical microbiology laboratories of three tertiary care hospitals in Thailand; 44 isolates were obtained from Thammasat University Hospital, 100 isolates from Siriraj Hospital, and 46 isolates from Songklanagarind Hospital. Thammasat and Songkhlanagarind isolates were obtained in April 2016 and August 2016, respectively, and the Siriraj isolates represent consecutive laboratory isolates cultured in April 2016. Virulent K. pneumoniae clinical isolate B5055 (serotype K2:O1) and capsule knockout derivative B5055nm (72) were kindly provided by Richard Strugnell, University of Melbourne.

Bacteria were identified by routine biochemical tests for identification of Gram-negative bacteria. Genomic DNA was extracted and sequenced using Illumina-B HiSeq X paired-end sequencing. Annotated assemblies were produced as previously described (73); sequence reads were assembled *de novo* using Velvet v1.2 (74) and either VelvetOptimiser v2.2.5 (75) or SPAdes version 3.10 (76) and annotated using PROKKA v1.11 (77). The stand-alone scaffolder SSPACE (78) was employed to refine contig assembly, and sequence gaps were filled using GapFiller (79). Contigs were annotated using PROKKA (77). Genomes with greater than 5% contamination levels as determined by Kraken (80), fully assembled genomes of 5 Mbp or less and 6 Mbp or more as well as those comprising 500 or more contigs were removed. Putative genomes with less than 60% sequence homogeneity with the reference genome were assessed with CheckM (81) for genome completeness and contamination; isolates with greater than 3% contamination levels were rejected. SNPs were called against the K. pneumoniae reference genome to identify heterozygous SNPs (Het SNPs) and isolates with greater than 2% Het SNPs were removed from further analysis (73), resulting in the 164 genomes analyzed in this study.

### Bioinformatic analyses.

The pan genome was determined with Roary (82), using a Protein BLAST identity of 95% and a core definition of 99%. SNPs were extracted from the core gene alignment using SNP sites (83) and the output used to run RAxML V8.2.8 (84) in order to calculate the phylogenetic tree with 100 bootstraps under the GTR time-reversible model. Antibiotic resistance genes were detected with the curated version of the ARG-ANNOT database available at the SRST2 site (85) using Ariba software (86). Genomes were assigned to STs by mapping to known alleles with SRST2 in accordance with the K. pneumoniae multilocus sequence typing (MLST) database (53). Virulence gene sequences were retrieved from the K. pneumoniae BIGSdb database at Institut Pasteur (http://bigsdb.web.pasteur.fr) and predicted using a custom-made Ariba database as described in the software manual (84). Plasmids were detected with the PLasmidFinder database (87) using Ariba software. Representations of trees and metadata were performed using iTOL (88) and the ggplot2 package in R (https://cran.r-project.org/web/packages/ggplot2/ggplot2.pdf). Kaptive (42) was used to determine capsule (K-type) and O-antigen (O-type) genotypes. Genes encoding OM proteins, components involved in sialic acid metabolism, and transport genes were investigated using customized Ariba databases.

### C′ susceptibility.

Susceptibility of K. pneumoniae isolates to commercial (MP Biomedicals UK) pooled human sera was determined essentially as described previously (32). The quality-controlled sera (https://www.mpbio.com/includes/msds/0929301/MP_COAT_0929301.pdf) were dispensed into small aliquots to avoid freeze-thaw cycles and stored at −80°C, and individual aliquots were thawed as required and used immediately following previously described recommendation (32). DS and S control K. pneumoniae isolates were employed during each assay run to ensure that storage conditions did not result in reduced levels of bactericidal potency. Briefly, early mid-logarithmic-phase Luria-Bertani (LB) broth cultures (200 µl) were washed three times in gelatin-veronal-buffered saline containing Mg^2+^ and Ca^2+^ (pH 7.35) (GVB^++^), suspended in 400 µl of GVB^++^, and mixed with 800 µl of prewarmed (37°C) serum to give a final concentration of ∼7.5 × 10^6^ CFU/ml. The mixtures were incubated at 37°C for 3 h, and bacteria were quantified by serial dilution and incubation on LB agar overnight. Prewarmed, heat-inactivated (56°C for 30 min) serum served as a control.

### Characterization of exopolysaccharide capsules.

The string test (17) was used to identify isolates producing hypermucoviscous capsular material. Strains were cultured on 5% sheep blood agar at 37°C overnight, and a standard bacteriological loop was employed to stretch a mucoid string from a colony: hypermucoviscosity was defined by the formation of viscous strings extending >5 mm in length. The surface area occupied by capsule was determined by mixing bacterial suspensions in PBS with an equal volume of India ink, applying to a microscope slide, attaching a coverslip, and obtaining photomicrographic images with a Zeiss Axiostar plus transmitted light microscope fitted with an Olympus SC30 digital camera and using a 100× oil immersion lens and embedded scale bar. Images were analyzed with CellProfiler image analysis software (89). Capsule content of selected isolates was also determined by precipitation of the polysaccharide followed by determination of uronic acid by modified carbazole (sodium tetraborate) assay (90, 91).

### Data availability.

Data have been deposited in the European Nucleotide Archive under accession no. ERP021210.
