# Supplementary figures and images for: Complement Susceptibility in Relation to Genome Sequence of Recent Klebsiella pneumoniae Isolates from Thai Hospitals
Source: mSphere. 2018 Nov 7;3(6):e00537-18. doi: 10.1128/mSphere.00537-18 (PMC6222052; doi:10.1128/mSphere.00537-18)

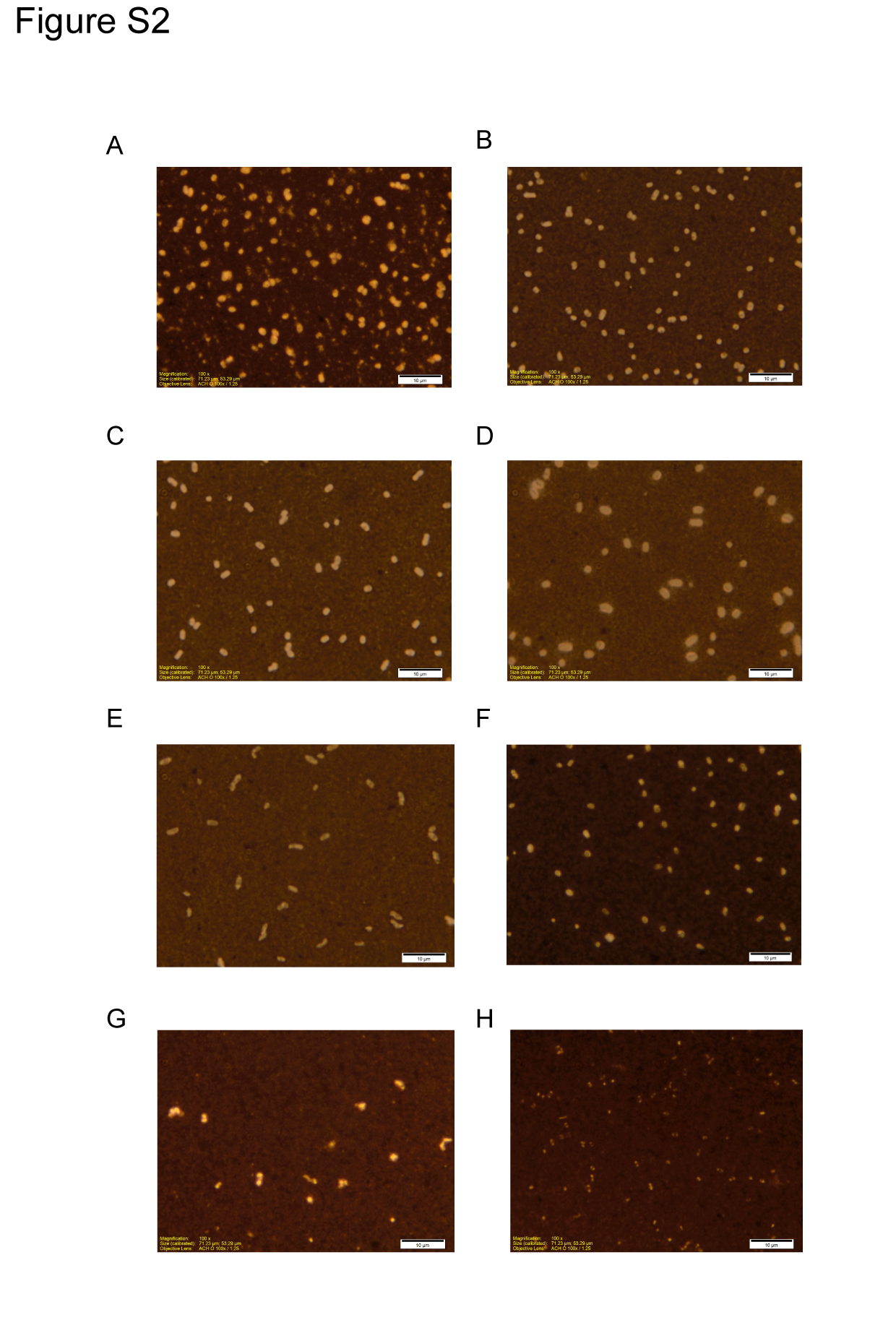

Supplement: FIG S2 [file sph006182700sf2.tif]

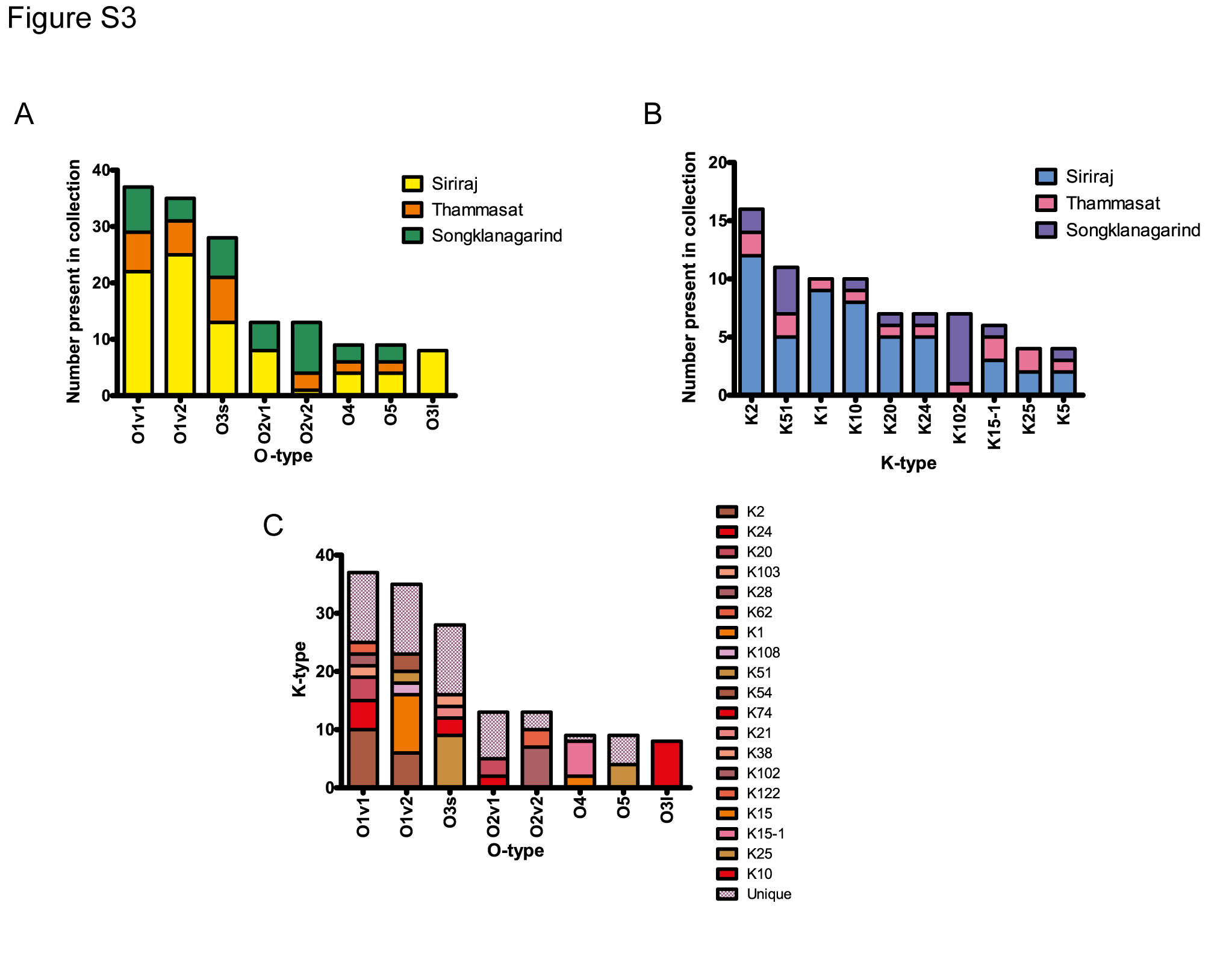

Supplement: FIG S3 [file sph006182700sf3.tif]

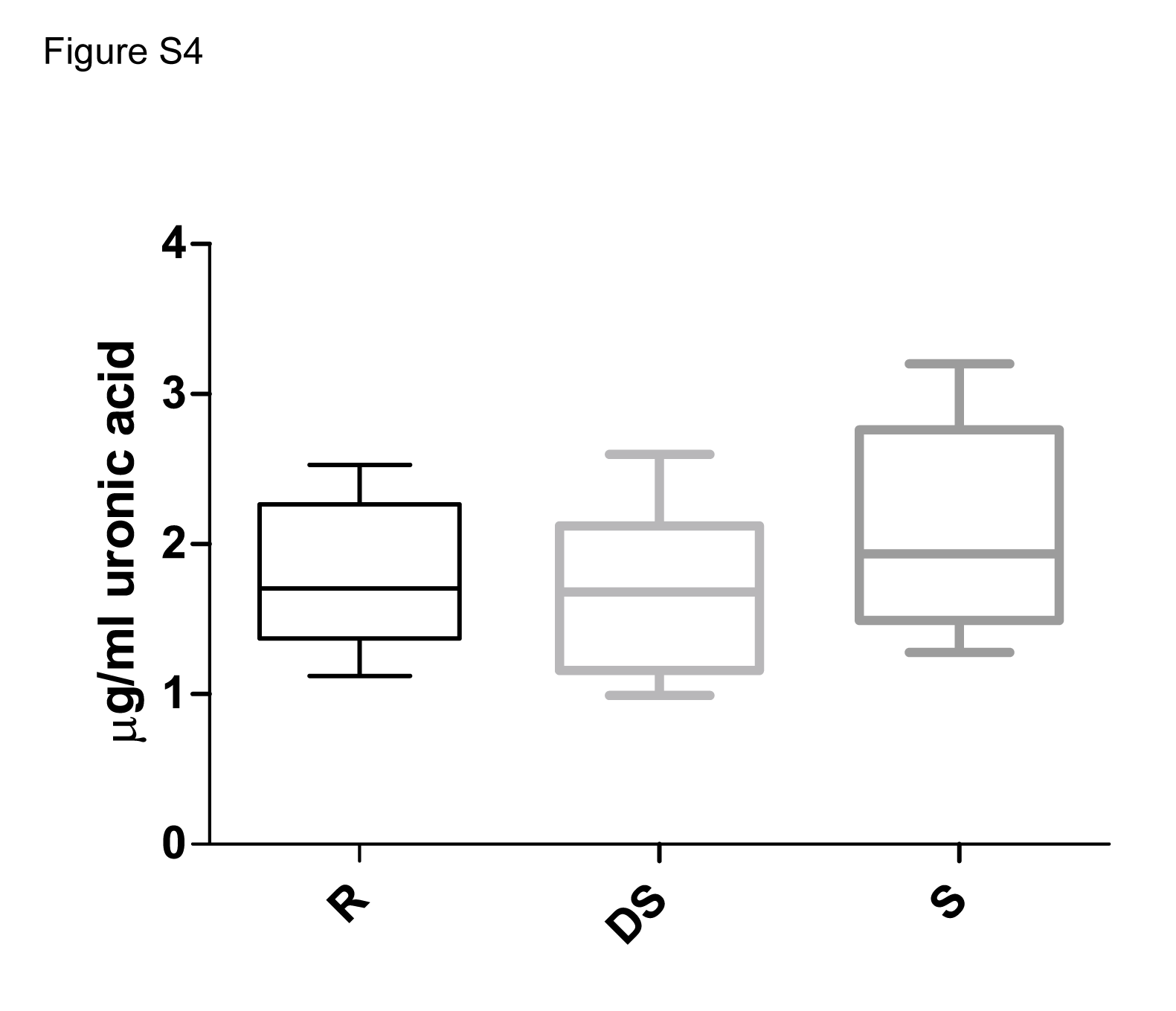

Supplement: FIG S4 [file sph006182700sf4.tif]
