# Supplementary material for: Complement Susceptibility in Relation to Genome Sequence of Recent Klebsiella pneumoniae Isolates from Thai Hospitals
Source: mSphere. 2018 Nov 7;3(6):e00537-18. doi: 10.1128/mSphere.00537-18 (PMC6222052; doi:10.1128/mSphere.00537-18)
Supplement: TABLE S1 [file sph006182700st1.docx]

**Table S1**

| **K Locus** | **R** | **DS** | **S** |
| --- | --- | --- | --- |
| **KL1** | 10 | 0 | 0 |
| **KL10** | 10 | 0 | 0 |
| **KL102** | 2 | 4 | 1 |
| **KL2** | 10 | 1 | 5 |
| **KL51** | 5 | 1 | 5 |
| **KL20** | 5 | 1 | 1 |
| **KL24** | 3 | 1 | 3 |
| **KL15-1** | 4 | 1 | 1 |
| **KL25** | 3 | 1 | 0 |
| **KL122** | 2 | 0 | 1 |
| **KL21** | 2 | 1 | 0 |
| **KL28** | 1 | 2 | 0 |
| **KL5** | 2 | 1 | 0 |
| **KL54** | 3 | 0 | 0 |
| **KL62** | 3 | 0 | 0 |
| **KL74** | 0 | 0 | 3 |
